# Experimentally induced intrasexual mating competition and sex‐specific evolution in female and male nematodes

**DOI:** 10.1111/jeb.13706

**Published:** 2020-10-01

**Authors:** Josefine Stångberg, Elina Immonen, Pilar Puimedon Moreno, Elisabeth Bolund

**Affiliations:** ^1^ Department of Ecology and Genetics, Animal Ecology Uppsala University Uppsala Sweden; ^2^ Department of Ecology and Genetics, Evolutionary Biology Uppsala University Uppsala Sweden

**Keywords:** *Caenorhabditis remanei*, experimental evolution, intralocus sexual conflict, life history, sexual dimorphism, trade‐off

## Abstract

Sexual dimorphism in life history traits and their trade‐offs is widespread among sexually reproducing animals and is strongly influenced by the differences in reproductive strategies between the sexes. We investigated how intrasexual competition influenced specific life history traits, important to fitness and their trade‐offs in the outcrossing nematode *Caenorhabditis* *remanei*. Here, we altered the strength of sex‐specific selection through experimental evolution with increased potential for intrasexual competition by skewing the adult sex ratio towards either females or males (1:10 or 10:1) over 30 generations and subsequently measured the phenotypic response to selection in three traits related to fitness: body size, fecundity and tolerance to heat stress. We observed a greater evolutionary change in females than males for body size and peak fitness, suggesting that females may experience stronger net selection and potentially harbour higher amounts of standing genetic variance compared to males. Our study highlights the importance of investigating direct and indirect effects of intrasexual competition in both sexes in order to capture sex‐specific responses and understand the evolution of sexual dimorphism in traits expressed by both sexes.

## Graphical abstract

Sex‐specific evolution of peak fitness in response to skewed sex ratios, with the ancestral (ANC) population compared to female‐biased (FB) and male‐biased (MB) treatment populations after 30 generations of experimental evolution in *Caenorhabditis remanei*. We found a significant interaction between sex and treatment, indicating sex‐specific evolutionary responses in peak fitness (λ_peak_). Thus, the increased potential for intrasexual competition in the common sex in each treatment leads to opposite responses in the two sexes.

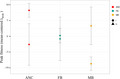

## 1 INTRODUCTION

Sexual dimorphism (*SD*) refers to phenotypic sex differences in traits other than sexual organs and can be manifested both as sex‐limitation of traits, and differential phenotypic expression of shared traits in the two sexes. *SD* is a widespread phenomenon in sexually reproducing taxa, found in a variety of traits, including morphological and life history traits as well as sexually selected ornaments and armaments (Shine, 1989; Wiens & Tuschhoff, 2020; Wyman et al., 2013). Phenotypic sex differences evolve in response to sex‐specific selection that stems from differences in reproductive strategies between the sexes (Fairbairn et al., 2007; Maynard Smith, 1976). For over a century, *SD* has been a target of intensive research to understand the evolution of intraspecific diversity (Andersson, 1994; Darwin, 1871; Wyman et al., 2013). The most obvious sexually dimorphic traits include conspicuous ornaments, common but not limited to males (such as the peacock's tail) (Amundsen, 2000; Nordeide et al., 2013), which has led to a historic focus on sexual selection on males (Andersson, 1994). Although the overall trend is that sexual selection is stronger on males, there is tremendous variation between taxa which we still need to understand better (Janicke et al., 2016), and how intense competition among females influences the evolution of sexual dimorphism is still poorly understood (Clutton‐Brock, 2007, 2009; Hare & Simmons, 2019). Studying sex‐specific selection simultaneously in both sexes is especially important for elucidating how dimorphism evolves in traits expressed in both sexes. The sexes are expected to compete in different ways due to their respective reproductive strategies and physiology and would hence be predicted to respond differently to increased intrasexual competition (Fisher, 1958). Moreover, many sexually dimorphic traits are correlated and involved in life history trade‐offs, and currently our knowledge of how sex‐specific selection operates on these trade‐offs through male–male and female–female competition is limited (but see Dale et al., 2015).

To study the evolutionary consequences of sex‐specific selection in species with two sexes, many previous studies have manipulated sex‐specific selection pressures, and the potential for intrasexual reproductive competition, by imposing different mating systems and typically comparing the ancestral state of multiple mating in both sexes with the removal of sexual selection and conflict via enforced monogamy (reviewed in Edward et al., 2010). Some studies have relaxed selection on one sex while maintaining it in the other, via certain breeding designs (Morrow et al., 2008). While there are many examples of such evolutionary manipulations in a variety of taxa (Bacigalupe et al., 2007; Crudgington et al., 2005, 2009; Fricke & Arnqvist, 2007; Holland & Rice, 1999; Linklater et al., 2007; Maklakov et al., 2009; Reuter et al., 2008; Tilszer et al., 2006; Wigby & Chapman, 2004), only few have altered sexual selection pressures and intrasexual competition simultaneously and analogously in both sexes. Edward et al., 2010 used skewed sex ratios (1:3 and 3:1) in *Drosophila melanogaster* and found sex‐specific responses both in terms of different traits responding to selection in the sexes, and some traits evolving in opposite directions within sexes between the treatments. Fritzsche et al. (2014) tested how primary reproductive traits evolve in each sex using slightly more skewed sex ratios (1:5 and 5:1) in an outcrossing nematode (*Caenorhabditis remanei)* and showed faster evolution in female‐specific traits under both increased male–male and female–female competition (Fritzsche et al., 2014). However, primary reproductive traits and mating traits, such as genital morphology and sperm plugs investigated in these two studies, are limited to one sex and are thus expected to respond more freely to sex‐specific selection than traits that are expressed by both sexes, which commonly have a shared genetic basis in the sexes (Lande, 1980; Parker, 1979). How traits expressed in both sexes evolve when experimentally increased strength of sexual selection is applied analogously on females and males has, to our knowledge, previously not been tested.

Sex‐shared traits include body size and life history traits that are often at the core of reproductive strategies in the sexes (Roff, 2002; Stearns, 1989). In addition to being genetically correlated between the sexes (Poissant et al., 2010), different life history traits are commonly also correlated with one another and embroiled in fitness trade‐offs (Gadgil & Bossert, 1970; Roff, 1993). Because trade‐offs among fitness‐related traits are often sex‐specific (Arak, 1988), selection can therefore alter life histories both through trade‐offs in the focal sex (Williams, 1957) as well as through correlated responses due to selection on the opposite sex (Lande, 1980; Poissant et al., 2010). For example, male‐limited selection on longer lifespan in a seed beetle (*Callosobruchus maculatus*) not only resulted in the evolution of other functionally integrated traits (metabolic rate, body mass and locomotor activity) in males but also to correlated multivariate evolution of the same traits in females (Berger et al., 2014). The selection for longer lifespan in males also revealed sexually antagonistic effects on fitness, decreasing male but increasing female fitness (Berg & Maklakov, 2012). Another study on *C. maculatus* similarly revealed sex‐specific life history evolution by showing how natural selection on age‐at‐reproduction, applied to both sexes, resulted in different responses in longevity in the sexes (Maklakov et al., 2009). In the nematode *C. remanei*, an increase in lifespan (with the drug Rapamycin) decreased the body size in both sexes but reduced fitness only in females, again suggesting a sex‐specific trade‐off (Lind et al., 2016). In contrast, a study on field crickets (*Teleogryllus commodus*) showed that while the reproductive effort was genetically correlated between the sexes in this species, the genetic correlation between reproductive effort and longevity differed between the sexes, resulting in a low genetic covariation for longevity (Zajitschek et al., 2007). These studies illustrate how sex‐specific trade‐offs are common but also variable across species and contexts, and highlight that we can expect that the response to sex‐specific selection will vary depending on how strong the genetic correlation is between individual traits, between the sexes and between the traits and fitness.

In this study, we investigated how intrasexual competition acts on different life history traits important to fitness and affect their trade‐offs, using an outcrossing nematode, *C. remanei*, as the model system. We altered the strength of sex‐specific selection through experimental evolution that increased the potential for intrasexual competition by strongly skewing the adult sex ratio towards females or males (1:10 or 10:1) over a period of 30 generations. We subsequently measured the evolutionary change in three phenotypic traits related to fitness; body size, fecundity and tolerance to heat stress. Importantly, we tested for evolutionary responses in both sexes in parallel when evolving under either competition regime, allowing us to examine how each sex responds directly as well as indirectly to increased sexual competition. We predicted that the skewed sex ratios will result in sex‐specific responses in life history traits and specifically that the more abundant sex is expected to show a stronger evolutionary response to altered strength of selection.

## 2 METHODS

### 2.1 Study species

We used the obligate outcrossing nematode species *Caenorhabditis remanei* that exhibits two separate sexes (i.e. gonochorism) and has a 50:50 sex ratio at birth. Importantly, both wild and laboratory populations show sexual dimorphism in key life history traits, such as lifespan, body size and timing of reproduction; females are larger than males, males live longer than females, and males are reproductively mature earlier and can reproduce for a longer period than females (Diaz et al., 2008, 2009). These life history properties as well as the short generation time (4 days) and the ease of laboratory rearing make *C. remanei* a highly suitable model organism for experimentally studying the evolution of sexual dimorphism.

A wild‐type strain (SP8‐50G) of *C. remanei* was used as our base population, here referred to as Ancestral (ANC). An additional advantage of using nematodes is that we can cryopreserve populations from months to years at −80°C and revive them by thawing, hence allowing us to compare the experimental evolution lines with the ancestral line simultaneously. This avoids the issue of additional differences occurring between then ancestral population and the treatment populations caused by drift sometimes associated with normal maintenance of lines in the laboratory over time. The strain (SP8) was originally created by crossing three wild‐type isolates, created by N. Timmermeyer at the Department of Biology at University of Tübingen, Germany (described in Fritzsche et al., 2014). The strain was subsequently cultivated at a large population size for 15 generations, before it was sent to us and cultivated for a further 30 generations as 6 replicated populations (Lind et al., 2020), which were then mixed and cultivated for another 5 generations at a large population size at the Department of Ecology and Genetics, at Uppsala University, Sweden. Hence, this strain was allowed to adapt to the laboratory environment for a total of 50 generations prior to the experimental evolution treatments (hence named SP8‐50G). The SP8 strain harbours plenty of standing genetic variation for life history traits (Chen & Maklakov, 2012), which is a prerequisite for experimental evolution, and the populations used to create the SP8‐50G strain are well adapted and have high fitness under standard laboratory conditions (Lind et al., 2020).

In the laboratory, the nematodes are kept on agar plates containing a lawn of bacterial food (*Escherichia coli*, strain OP50), and the plates are kept in climate cabinets at 20°C, with 60% humidity and under dark conditions. Worms were handled under the microscope (Leica M165C stereomicroscope) and handling times were minimized and standardized across populations as far as possible to equalize exposure time to the conditions outside the climate cabinet. Populations were cryopreserved, and kept at −80°C until thawed and used for assays, according to standard protocol (Brenner, 1974; Girard et al., 2007).

### 2.2 Experimental evolution

Experimental evolution is a method to study the evolution of traits in real time (Garland & Rose, 2009). Populations evolve under set conditions for a number of generations. At the end of the experimental evolution process, populations are placed in a common garden environment for 2–4 generations, to minimize any transgenerational parental effects or phenotypic plasticity. Hence, the phenotypic differences apparent between the base population and the individuals from experimentally evolved populations can be attributed to evolutionary responses to selection, imposed by the specific treatments (Garland & Rose, 2009; Kawecki et al., 2012; Sikkink et al., 2014). Evolutionary changes may, however, be due to random processes such as inbreeding and drift, which may be especially important in the limited population sizes that are often required for logistic reasons in experimental evolution studies (Snook et al., 2009). To limit the effect of drift, we used the base population (SP8‐50G, described above) that has a high level of genetic diversity, as well as making sure the treatment populations maintained an effective population size (N_e_) of approximately 100 (see details below). Additionally, the use of three replicate populations for each treatment allows us to account for the effects of random processes which may occur over time in during the experimental evolution.

We used skewed adult sex ratios to create environments with increased potential for intrasexual competition. The sex ratios used were 1:10 and 10:1 (male:female) and are referred to as female‐biased (FB) and male‐biased (MB) treatments, respectively. Populations were kept at these sex ratios for 4 days of adulthood, and the offspring for the next generation were collected on day 4 using a standardized bleaching protocol which age‐synchronized offspring, by limiting the surviving individuals only to eggs that are approximately 18‐20hr old (Stiernagle, 2006). This resulted in offspring from matings that took place during day 3 and 4 of adulthood, which are the peak days of reproduction in both sexes (Diaz et al., 2008). This was done to limit any unintentional selection for early reproduction. During this adult stage, each population was housed in 9 petri dishes (60 mm in diameter), each dish housing 3 individuals of one sex and 30 individuals of the other sex. Hence, each population was maintained with an actual population size (*N*) of 297, which corresponds to an effective population size (*N_e_*) of approximately 100 after accounting for the skewed sex ratios. (Falconer & Mackay, 1996). All eggs collected, and used for the next generation from each population, were mixed and allowed to develop on large plates (90 mm in diameter). We sexed the larvae two days after hatching, before they reach sexual maturity (larval stage L4), and randomly chose individuals to transfer to a new set of 9 plates. The experimental evolution was maintained for 30 generations, with 3 replicate populations per treatment (FB and MB). At the end of the experimental evolution, populations were cryopreserved. Treatment populations were subsequently compared to the base population SP8‐50G, referred to as the ancestral population (ANC).

### 2.3 Phenotypic assays

Worms were taken from cryopreservation for assays. The full process of cryopreservation includes 4 generations in a common garden, 2 generations when freezing them and 2 generations when thawing them for assays. The ancestral population (ANC) and three replicate populations from each of the two treatments (FB and MB) were assayed, a total of 7 populations. For each treatment, a total of 720 individuals were assayed; hence, the same number of individuals was assayed for ANC, FB and MB. From each replicate population, 240 individuals (120 females and 120 males) were assayed. For logistical reasons, one assay block was limited to 120 individuals (60 individuals of each sex). Thus, each replicate population was assayed twice and the ancestral population 6 times, resulting in 18 assay blocks in total. For each focal nematode, three phenotypic traits were measured: body size, offspring production and heat shock tolerance.

#### 2.3.1 Body size

All focal individuals, males and females, were assayed for body size on day 3 of adulthood, the first day of peak reproduction. Pictures were taken individually on plates in a microscope with a mounted camera (Leica M165C stereomicroscope, Lumenera Infinity 2‐5C digital microscope camera). We also photographed females on day 6 of adulthood, because previous studies have found that female body size after peak reproduction (days 3 and 4 of adulthood) may change, by continued growth or shrinkage, and this could potentially be different between the ancestral population and the treatment populations (Lind et al., 2016; Shi & Murphy, 2014). The microscope pictures were analysed using Fiji (ImageJ http://imagej.nih.gov/ij/) plugin called Wormsizer (Moore et al., 2013), where total body volume (in cubic picometre, pm^3^) was used as a body size measurement.

#### 2.3.2 Offspring production and peak fitness (λ_peak_)

Reproductive output was measured as the total number of offspring produced on each day in a noncompetitive assay, for the first 7 days of adulthood, for each individual. The measurements for males and females differ, as described below, because of biological sex differences. Each focal individual was given a standard partner from the base population: an individual unmated worm of standardized age (day 1 of adulthood) of the opposite sex. The focal individual was given a new standard partner daily during the 7 days of the reproductive assay. For statistical analysis, we used a weighted measure of peak fitness, (*λ*_peak_), giving a reproductive value based on offspring production on days 3 and 4 of adulthood (peak reproduction), and weighting early reproduction (day 3) heavier than late reproduction which fits well the nematode reproductive pattern (Brommer et al., 2002; Lind et al., 2016). This measurement also captures the experimental evolution time frame, since offspring were selected from matings on days 3 and 4 only. This value was calculated separately for males and females, but because male reproductive values are much higher than females, we used mean‐centred peak fitness (*λ*_peak_) to be able to compare the treatments across the sexes on the same scale. Results were qualitatively consistent when reproduction was alternatively analysed, both using daily reproduction in repeated measures models, and using lifetime reproductive success (LRS) (not shown). In addition to the overall effect of treatment and the interaction between sex and treatment, we were also interested in the effect of body size on *λ*_peak_, and its sex‐specific importance. We therefore added mean‐centred body size as a covariate in the models of peak fitness (*λ*_peak_).

##### Female offspring production

**FIGURE 1:**
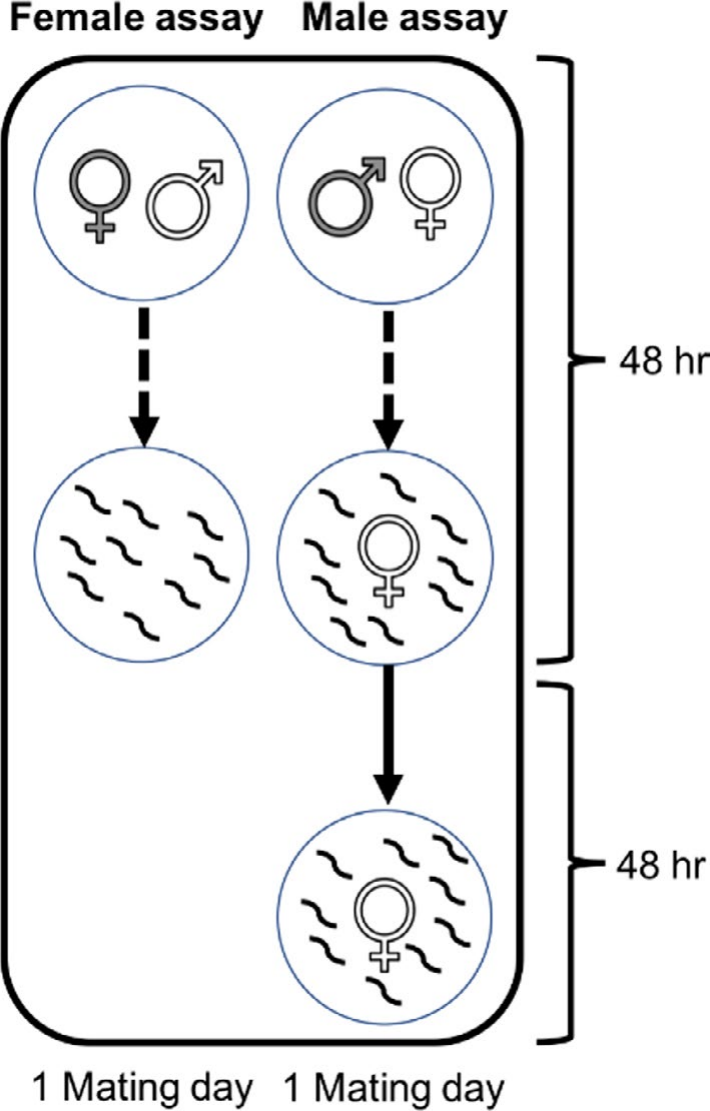
Comparison between female and male offspring production assays from one mating day, the female assay on the left and the male assay on the right. The focal sex is in dark grey, and the standard partner in white. In the female assay, the focal female was placed with a male standard partner and allowed to mate and oviposit for 24 hr. After this, the standard male partner was removed, and the focal female was moved to a new mating plate with a new standard mating partner. For the male assays, the focal male was also moved to a new mating plate after 24 hr of mating, but the standard female partner was kept an additional 24 hr on the mating plate for egg laying and subsequently transferred to a second plate to continue to lay eggs for an additional 48‐hr period. Hence, the standard female partner was able to lay all eggs fertilized during the 24‐hr period spent with the focal male. Due to the differing reproductive physiology and the logistics that follow, the male mating assays generated a higher total offspring number per mating day

On each of the 7 days, at the same time of day each day, the focal female was moved to a new plate with a novel partner. The previous standard male partner was discarded, and this plate was kept for an additional 24 hr, in the climate cabinets, for offspring to hatch and grow. The total count of offspring on each plate corresponds to daily female offspring production, Figure 1.

##### Male offspring production

Each day at the same time, the focal male was moved to a new plate with a new female partner. Pilot studies showed that females under these conditions continue to lay eggs during approximately 72 hr after separation from the partner (personal observation). To give a complete picture of the total number of eggs fertilized by the focal male, in the 24‐hr period, the female partner thus remained on the old plate, to allow egg laying for an additional 24 hr, followed by transfer to a new plate to avoid offspring starting to reproduce on the plate. An additional 48 hr of egg laying was allowed before the female was discarded (96 hr in total). The total number of offspring produced during the 96 hr corresponds to male success in stimulating female fecundity and fertilizing the eggs, Figure 1.

#### 2.3.3 Heat shock tolerance

We used heat shock in this study to test for differences in stress tolerance as a proxy for somatic maintenance (Amrit et al., 2010). A heat shock was triggered by transferring the worms at day 8 of adulthood (after the reproductive assay was concluded) into a cabinet with a temperature of 37.5°C, which is stressful compared to their usual temperature of 20°C (Mautz et al., 2020). The worms were then tested for heat shock tolerance, which was done by briefly removing them from the climate cabinet and very lightly poking each worm once an hour, for 7 hr, using a thin platinum wire to check for responsiveness. When the worms reached the level of unresponsiveness where they were no longer able to freely move away from their spot on the plate when poked, they were scored as having reached the event “death” (class C according to Herndon et al., 2002). The value used for analysis is the number of hours of heat shock until “death,” which is analysed using survival analysis. Heat shock tolerance corresponds well with lifespan and thus functions as a proxy for somatic maintenance in this study system (*C. remanei,* Amrit et al., 2010). For analytical purposes, the individuals that reached “death” within the first three hours of heat shock are referred to as “sensitive” (S), and the worms that reached “death” after 4–7 hr, or not at all, are referred to as “robust” (R).

### 2.4 Statistical methods

We analysed body size and reproduction data with linear mixed‐effect models in the *lme4* package (version 1.1‐23) in *R* (R Core Team, 2018) (Bates et al., 2015) and the statistical significance of effects were estimated using ANOVA tests implemented in the *lmerTest* package. Heat shock tolerance data were analysed with mixed effects cox models using the R packages *survival* (version 3.1‐8) (Therneau & Grambsch, 2000) and *coxme* (version 2.2‐16) (Therneau, 2020). All models accounted for block (18 levels) and population (7 levels) as random effect factors, with block nested within population and population nested within treatment. The focal individuals of the assays were the offspring of a quantitative genetic breeding design (for purposes of another manuscript, in preparation). To account for this, all models were run with sire and dam as random effects, with dams nested within sires. Reproduction models additionally contained body size as a covariate, to account for the positive relationship between body size and fecundity that is commonly assumed (Roff, 2002; Stearns, 1989). Since both body size and peak fitness measures differ greatly between males and females in our study (females being much larger, males having much higher peak fitness), we used mean‐centred data to be able to make treatment comparisons across the sexes. However, for the direct sex comparisons of size, nonstandardized data were used. Mean centring of data is a minimally invasive standardization, which sets the mean for each sex to zero, but leaves the variance of the data unchanged. We were mainly interested in the overall effect of treatment and the interaction between sex and treatment for all the traits measured. Although the sample sizes were 720 individuals (per treatment) at the beginning of the assays, the actual sample sizes are somewhat lower due to deaths occurring during assays (see Appendix S1 for actual sample sizes). The statistical methods used in this study are robust to unbalanced designs.

We checked the model performance by visually inspecting residual distributions for non‐normal distributions and heteroscedasticity. All analysis were conducted using *R* (version 3.6.3) (R Core Team, 2018) and *RStudio* (version 1.2.5042) (RStudio Team, 2015).

## 3 RESULTS

### 3.1 Body size

**FIGURE 2:**
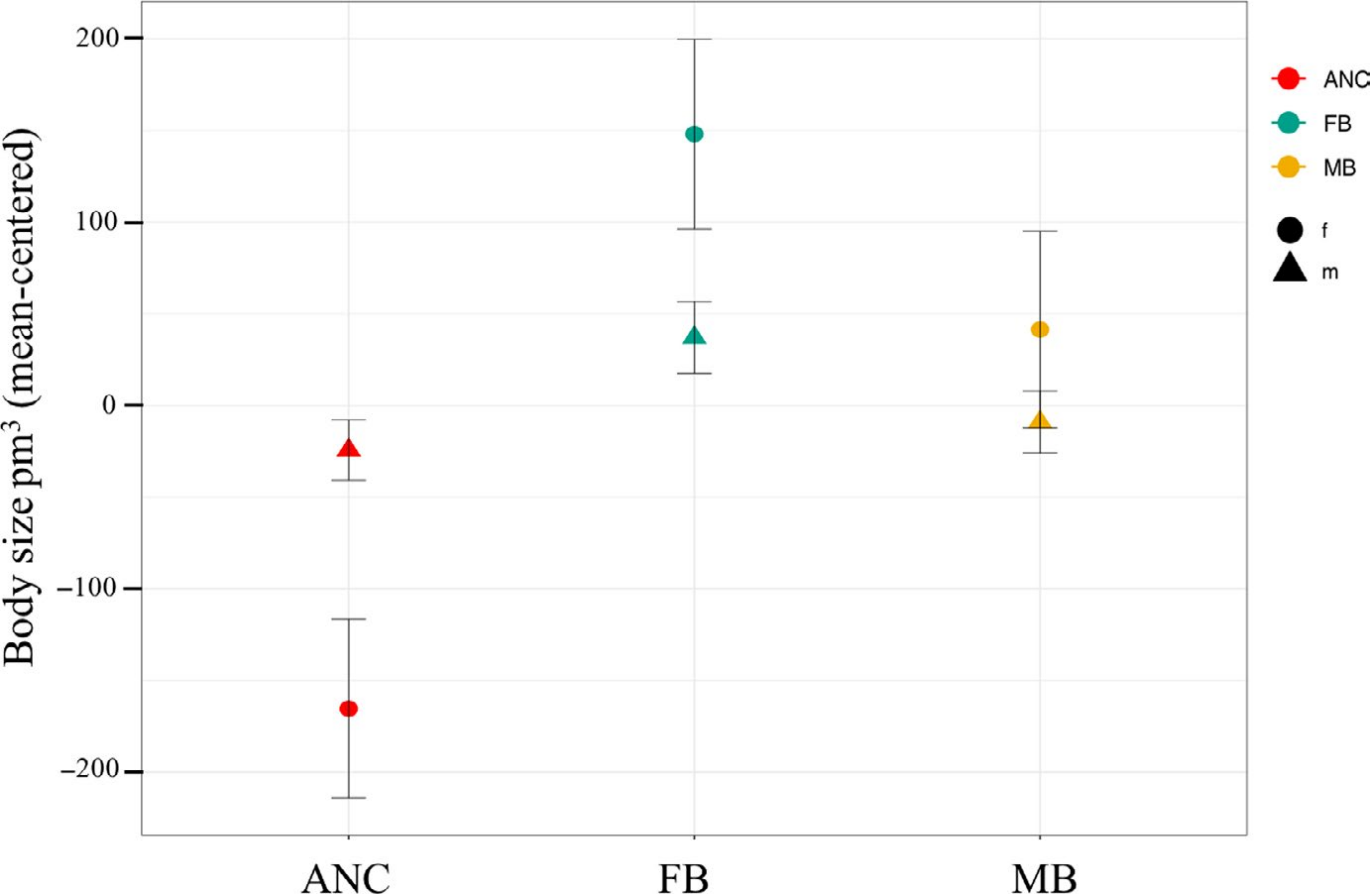
Within sex comparisons of treatment differences in body size, on day 3 of adulthood. The three treatments are shown on the x‐axis, and the within sex mean‐centred data on the body size (treatment means with standard errors) on the y‐axis. In red is the ancestral (ANC) population, in green female‐biased (FB) populations and in yellow the male‐biased (MB) populations. Circles represent females and triangles represent males

The results from our linear mixed‐effect model (of the nonstandardized data) show a clear and expected sexual dimorphism in body size, across all three population types (ANC, MB and FB), with males being significantly smaller than females (mean body size on day 3 of adulthood for females: 4574 ± 30.42 pm^3^, males: 1596 ± 9.91 pm^3^, *F*_1,2_ = 10,870, *p* < .001). Our analysis (using the mean‐centred data) found no overall effect of treatment on body size (*F*_2,1_ = 0.79, *p* = .56); however, there was a significant interaction between sex and treatment (*F*_2,2_ = 8.12, *p* < .001), suggesting that the two sexes have responded differently to the experimental evolution treatments when compared to the ancestral state (Figure 2.). In females, there was also a significant effect of day of adulthood on body size, whereby all females (regardless of population) grew to be significantly larger on day 6 compared to day 3 (*F*_1,2_ = 1,499, *p* < .001); hence, peak body size occurs sometime after peak reproduction. For details, see Appendix S1.

### 3.2 Peak fitness (*λ*_peak_)

**FIGURE 3:**
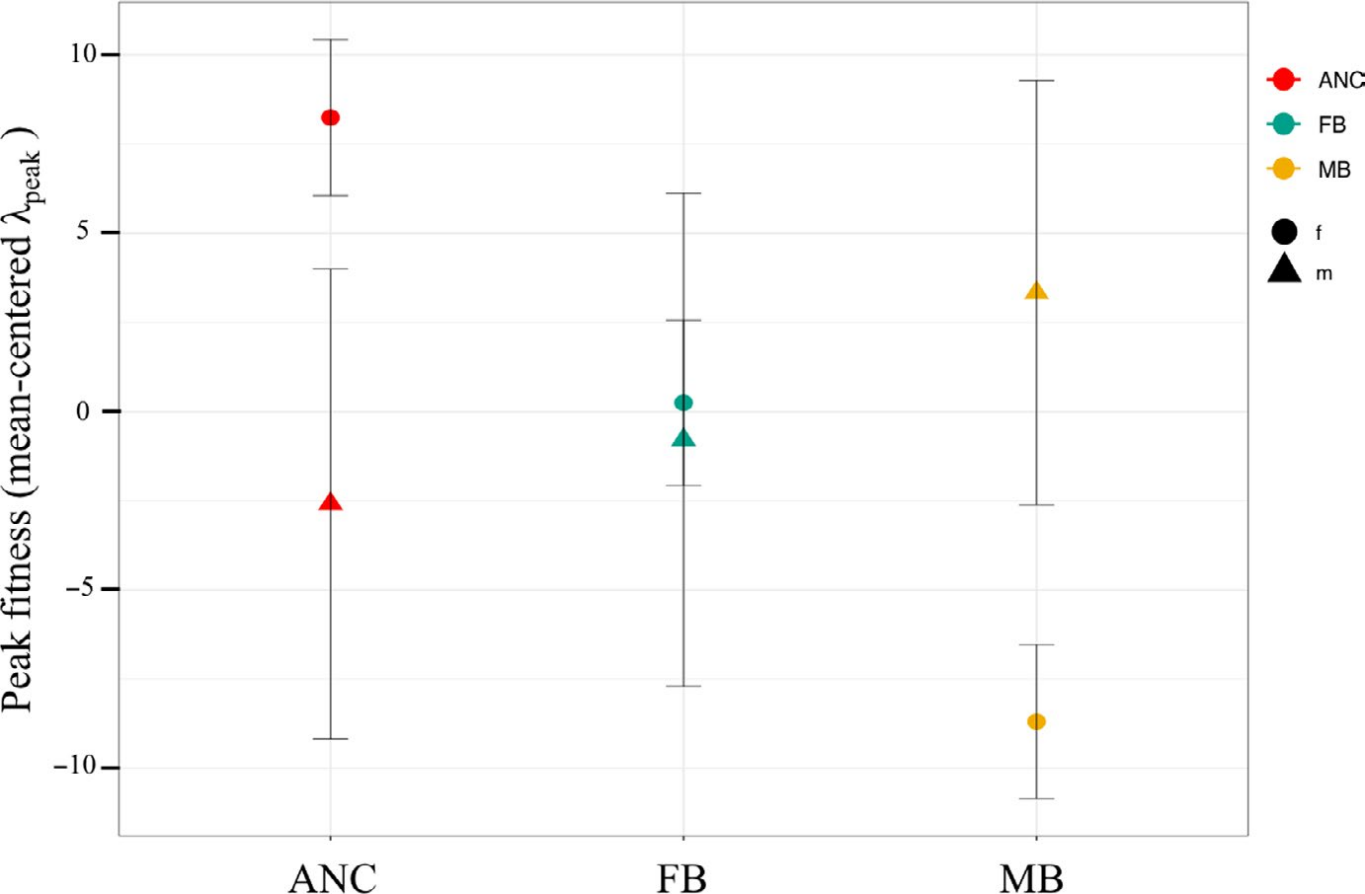
Within sex comparisons of peak fitness (λ_peak_) (treatment level means with standard errors) for in all three treatments. The three treatments are shown on the x‐axis, and mean‐centered (within sex) peak fitness (λpeak) on the y‐axis. The ancestral (ANC) population in red, female‐biased (FB) populations in green and male‐biased (MB) populations in yellow. Circles represent females, and triangles represent males. There is a significant interaction between treatment and sex, suggesting the sexes have responded differently depending on which sex was subjected to increased intrasexual competition

Our analysis shows no significant overall effect of treatment on peak fitness (*λ*_peak_) (*F*_2,1_ = 0.12, *p* = .89). There is, however, a significant interaction between treatment and sex (*F*_2,1_ = 3.75, *p* = .024, Figure 3) suggesting that the sexes have responded differently depending on which sex was subjected to increased intrasexual competition. Females from the treatment populations tend to have a lower *λ*_peak_ compared to ANC females. In contrast, males from treatment populations, tend to have a slightly higher *λ*_peak_ compared to ANC males. This effect is driven by the reversal of the MB treatment effect on the sexes (post hoc test, *t*‐value = 2.73, *p* = .00649), explaining the overall treatment‐by‐sex interaction. Additionally, there was a significant effect of body size on peak fitness (*λ*_peak_) (*F*_1,2_ = 31.68, *p* < .001), where a larger size is associated with increased fitness, and this effect was stronger for females (*F*_1,1_ = 12.71, *p* < .001, *p*_adjusted_ < 0.001) than for males (*F*_1,1_ = 3.31, *p* < .001, *p*_adjusted_ = 0.002). For details, see Appendix S1.

### 3.3 Heat shock tolerance

**FIGURE 4:**
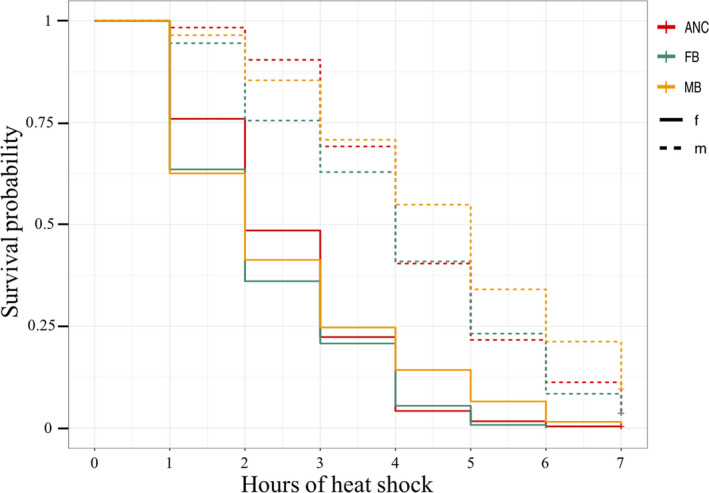
Survival probability of the nematodes exposed to heat shock treatment. The x‐axis shows the number of hours of heat shock, on the y‐axis is the proportion alive. Dashed lines represent males, full lines represent females. In red is the ancestral (ANC) population, in green female‐biased (FB) populations, and in yellow male‐biased (MB) populations

Males are overall more robust compared to females when subjected to heat shock, regardless of treatment (Exp_malesS_ = 0.61 ± 0.14, *z*_malesS_ = −3.48, *p* < .001 and Exp_malesR_ = 0.39 ± 0.17, *z*_malesR_ = −5.76, *p* < .001, where S refers to sensitive individuals, and R refers to robust individuals). We found no support for an interaction between treatment and sex. Kaplan–Meier survivorship curves (Figure 4) show that survival curves of the treatment populations cross the survival curves of the ancestral population in both sexes after 3 to 4 hr of heat shock, thus violating the proportional hazards assumption. To account for this, differences in heat shock tolerance between the ancestral and treatment populations can be assessed separately in individuals that were “robust” (R) and “sensitive” (S) (Table 1, Figure 4), thus fulfilling the proportional hazards assumption within each time period (Kleinbaum & Klein, 2012). The (S) group was overrepresented in the FB treatment, but not the MB, compared to the ancestral population (Exp_FBS_ = 1.4 ± 0.16, *z*_FBS_ = 2.2, *p* = .029 & Exp_MBS_ = 1.3 ± 0.16, *z*_MBS_ = 1.8, *p* = .079), in both sexes. Conversely, (R) individuals were more common in the male‐biased (MB) treatment compared to the ancestral population (Exp_MBR_ = 0.53 ± 0.25, *z*_MBR_ = −2.5, *p* = .012). However, the proportion of (R) individuals in the female‐biased (FB) treatment did not significantly differ from the ancestral population (Exp_FBR_ = 1.07 ± 0.26, *z*_FBR_ = 0.25, *p* = .81). Models with body size as a covariate showed that body size has no significant effect on heat shock tolerance (all *p* > .05).

**TABLE 1:** The full coxme output for “Sensitive” (S) and “Robust” (R) individuals separately, comparing the female‐biased (FB) and the male‐biased (MB) populations with the ancestral (ANC) population

	Coef	Exp (coef)	*SE*	*Z*	*p*
“Sensitive” S (*n* = 645)					
Sex	−0.488	0.614	0.139	−3.48	<.001
Trt (FB)	0.352	1.422	0.161	2.18	.029
Trt (MB)	0.284	1.329	0.162	1.75	.079
“Robust” R (*n* = 809)					
Sex	−0.950	0.387	0.165	−5.76	<.001
Trt (FB)	0.063	1.065	0.257	0.25	.810
Trt (MB)	−0.638	0.528	0.253	−2.52	.012

Note

Standard errors (*SE*) are on treatment level.

## 4 DISCUSSION

The change in strength of selection, caused by a strong bias in the adult sex ratio for 30 generations, resulted in sex‐specific evolution of body size and reproductive fitness, revealed by the significant interactions between the evolutionary treatments and sex. Our results show a greater evolutionary change in females than males in these traits, in line with a previous study on the same species (Fritzsche et al., 2014), challenging the widely held assumption of stronger net selection on males (Agrawal, 2001; Janicke et al., 2016; Lorch et al., 2003; Siller, 2001). In contrast, we found no sex differences in the treatment effects on heat stress tolerance, suggesting that selection has acted similarly on somatic maintenance in the sexes.

Females evolved a larger body size under both sex‐ratio treatments, but particularly so under the female‐biased sex ratio, whereas males showed qualitatively similar but smaller changes. Despite their larger size, a comparison of peak fitness (*λ*_peak_) revealed a reduction in female fecundity under both evolutionary treatments relative to the ancestors. Although we observed that body size and fecundity are positively related, the evolutionary responses suggest a trade‐off between investing in a larger size (and its maintenance) and fecundity, or at least reduced fecundity selection (Fitzpatrick et al., 1995; Hare & Simmons, 2019). Under the female‐biased sex ratio, this could occur if females become sperm‐limited in an environment where males are rare and unable to fertilize all of their eggs (Dewsbury, 1982). Hence, female fitness may be more limited by the investment into mate competition through a higher mating rate and/or monopolization of males, which would be successful in a competitive environment but could come at the cost of investment into increased fecundity. This investment into competitively successful traits would not be found in a noncompetitive assay (as in our study), but the cost to fecundity investment would. There are examples from other species where large females are observed performing more competitive behaviours towards other females, than smaller females (e.g. Stuart‐Smith et al., 2007), and previous documentations of female body size evolving in response to female competition for mates (e.g. Berghe & Gross, 1989). Given that *C. remanei* females are sperm‐limited and unable to get all their eggs fertilized with sperm from a single mating (Diaz et al., 2009), a scenario where female body size has increased to improve mate competition ability under the female‐biased treatment is probable. Interestingly, towards the end of the experimental evolution process females from this treatment were observed to aggregate in high numbers around males (personal observation), a behaviour typically only seen in males. This behaviour could be important when competing for mates in both sexes and may thus select for the larger size. It is also possible that traits other than body size are important to competing females that we did not measure in our assays, such as behavioural traits or genital morphology (Cain & Ketterson, 2013; Fritzsche et al., 2014b). While males showed less changes than females, their slightly larger size under female‐bias could indicate a fecundity or sperm production benefit to males in a sperm‐limited environment, in line with a positive association between male size and fitness in our data. Alternatively, the subtle response in male size could be a correlated response to strong selection on females, as a result of a shared genetic basis of body size between the sexes commonly observed across taxa (Castillo, 2005; Fairbairn et al., 2007).

The sex‐specific selection pressures resulting from our sex‐biased treatments also revealed sexually antagonistic effects on fitness under a male‐biased sex ratio, due to a reduction in female fecundity and increase in male fecundity relative to the ancestors. This indicates a sexual conflict over male adaptations to sexual competition and female reproductive investment. Intralocus conflict occurs when selection on one sex displaces the other from its phenotypic optimum, due to a shared genetic basis for traits underlying fitness (Bonduriansky & Chenoweth, 2009; Doorn, 2009; Lande, 1980; Pennell et al., 2016; Rice, 1984). The change in males was however more subtle than in females and may not suffice to explain the reduction in female fecundity. Alternatively, this could result from an evolved trade off due to an investment in a larger body size, driven by inter‐locus sexual conflict (Schenkel et al., 2018). In *C. remanei,* females suffer reduced survival when faced with a large number of males(Diaz et al., 2009). It is possible that survival selection favours larger female size and somatic maintenance under male sexual harassment, which could reduce investment into fecundity due to a trade‐off. Male‐biased sex ratio may have also introduced a conflict over female fecundity. Increasing number of studies have shown that male sperm competition success depends not only on sperm quality investment but also on male ability to manipulate female egg laying rate (Alonzo & Pizzari, 2010; Cameron et al., 2007; Parker & Pizzari, 2010; Pascoal et al., 2018; Perry et al., 2013), commonly achieved through nonsperm components in the seminal fluid (Hopkins et al., 2017; Perry et al., 2013). Males from the male‐bias treatment could therefore be better at female fecundity stimulation, whereas females may have evolved a lowered sensitivity to this manipulation in order to maximize their net benefits from polyandry under high mating frequency. The fitness assays used here tested for the lifetime reproductive potential in a noncompetitive context, which could also mask some differences between treatments that only occur in direct response to competition. Additionally, as individuals were tested together with partners from the ancestral population, our assays will not detect changes that only occur in response to coevolved interactions between the sexes (e.g. reciprocal crosses, see Palopoli et al., 2015).

In contrast to the patterns in body size and fitness, we did not detect any sex differences in the way the heat shock tolerance evolved under the sex ratio treatments, despite the fact that males were overall more tolerant to heat stress than females, confirming earlier studies (e.g. Reynolds & Phillips, 2013). Our analysis revealed that the populations evolving under male‐biased sex ratio had a larger proportion of individuals more robust to heat stress (“robust,” Table 1), whereas those evolving under a female‐biased sex ratio had a larger proportion of less stress tolerant individuals (“sensitive,” Table 1). However, in the group of sensitive individuals the male‐biased treatment showed a higher mortality than the ancestral population, suggesting that the evolution under intense male competition resulted in overall increased variation in stress tolerance. Here, we used heat shock tolerance as a proxy for somatic maintenance (Amrit et al., 2010). The somatic cost of maintaining larger bodies is thought to be greater than investment into fecundity in many nematodes, leading to a trade‐off between body size and somatic maintenance (Keymer & Read, 1991; Morand, 1996). Investing in a larger body could thus explain why females are less tolerant to heat stress than males, but also why the stress tolerance was relatively lower for the individuals evolved under the female‐biased treatment. However, in our analysis we did not find any significant effect of body size on heat shock tolerance. This suggests that although females are larger than males, males have longer lifespan, and the investment into somatic maintenance translates into both longer lifespan and better heat shock tolerance rather than heat shock affecting larger bodies more severely (Kirkwood, 1977). Hence, in our populations it seems that body size is not the main cause of differences in heat stress tolerance between the sexes.

The majority of studies where sex ratio manipulation is used have focused on males, whereas very few studies have manipulated the sex ratio simultaneously in both sexes. In the few studies that have similarly manipulated intrasexual competition (e.g. Fritzsche et al., 2014), stronger response to selection in females has been observed, as was found in our study. Indeed, there has been an increase in interest towards understanding how competition and aggression evolves and manifests in females (e.g. in Rosvall, 2011), and a growing body of evidence shows that females can be under strong selection to achieve matings (Clutton‐Brock, 2007, 2009; Hare & Simmons, 2019; Rosvall, 2011; Shuker, 2010; Stockley & Bro‐Jørgensen, 2011). Females are predicted to compete under female‐biased sex ratios when males are limited (Emlen & Oring, 1977; Kvarnemo & Ahnesjo, 1996), and empirical work supports this (Forsgren et al., 2004; Kvarnemo et al., 1995). It is common for females, however, not to get fully excluded from mating when faced with increased competition for mates, especially in polygynous mating systems with a high male mating rate (Brown, 1969), which is the case for *C. remanei* (Diaz et al., 2008). Although using a strongly skewed sex ratio (1:10) greatly increase variation in mating success for the common sex, it is not necessarily so that the selection intensity in the competing sex will be equal in the two treatments.

A strong skew in the sex ratio, such as used in this study, is extreme but can readily happen in nature, for example in systems with male‐killing meiotic drivers (e.g. Jiggins et al., 1998) or in species with temperature‐dependent sex determination (e.g. Janzen, 1994). Additionally, as in many other systems, in *C. remanei* there is potential for a temporal skew in the operational sex ratio (OSR) (Emlen, 1976; Emlen & Oring, 1977), even when offspring sex ratio is 50:50, because males mature sexually earlier and remain reproductively active longer than females (Diaz et al., 2008). Coupled with overlapping generations, this can intensify sexual selection on males (Kokko & Monaghan, 2001). Importantly, factors other than the OSR will also influence the direction of sexual selection, such as advantages of mating with a high‐quality mate (Clutton‐Brock & Parker, 1992; Johnstone et al., 1996; Kvarnemo & Simmons, 1999; Owens & Thompson, 1994) and mortality differences (Tershy & Croll, 2000). Although OSR has been found to predict sexual competition well, it is difficult to disentangle the effects of OSR from such factors as quality mate choice and mortality differences (Kvarnemo & Ahnesjo, 1996). In *C. remanei*, however, where the adult lifespan is short in both sexes, and there is a high cost of reproduction, the large risk of not reproducing at all if mates become scarce and intrasexual competition is increased is expected to result primarily in the evolution of mate acquisition strategies (Kokko & Monaghan, 2001). Thus, the differences in intrasexual competition should be the main selection pressure causing changes in the treatment populations for the focal sex. Importantly, however, our results indicate that the direction of sex ratio bias does not necessarily predict which sex shows more phenotypic evolution. This finding is in line with the current view that the dynamics of sexual interactions result in sex‐specific selection on both sexes and not only on the sex that experiences strong competition for access to mates (Arnqvist & Rowe, 2005; Clutton‐Brock, 2007, 2017; Connallon et al., 2010).

## 5 CONCLUSIONS

In summary, our study shows how increased intrasexual competition results in sex‐specific evolution of life history traits and their trade‐offs in the two sexes, reflecting their different reproductive strategies. Females evolved greater body size and fecundity differences under both strong female and male sexual competition, suggesting that females may have experienced stronger net selection and potentially higher amounts of standing genetic variance. Our study highlights the importance of investigating both the direct effects of sexual competition, as well as their indirect selective consequences, on both sexes, in order to capture sex‐specific responses and understand the evolution of sexual dimorphism in sex‐shared traits.

### PEER REVIEW

The peer review history for this article is available at https://publons.com/publon/10.1111/jeb.13706.

## Supporting information

Appendix S1
