# Supplementary material for: Experimentally induced intrasexual mating competition and sex‐specific evolution in female and male nematodes
Source: J Evol Biol. 2020 Oct 1;33(12):1677–88. doi: 10.1111/jeb.13706 (PMC7756511; doi:10.1111/jeb.13706)
Supplement: Supplementary file 1 — Appendix S1 [file JEB-33-1677-s001.docx]

**Supplementary material**

**Table legends**

***Table 1*** *The Lmer output for body size (centered within sex) showing the effect of treatment and the interaction between treatment and sex.*

***Table 2*** *The Lmer output for female body size (pm^3^) on day 3 & 6 of adulthood, showing the effect of treatment, day and the interaction between treatment and day.*

***Table 3*** *Mean body size (pm^3^) with standard errors and actual sample sizes per sex, treatment and replicate population*

***Table 4*** *The Lmer output for peak fitness λ_peak_ (centered within sex) showing the effect of treatment and body size on peak fitness, as well as the interaction between treatment and sex.*

***Table 5*** *Mean peak fitness* (λ_peak_) *with standard errors and actual sample sizes per sex, treatment and replicate population*

**Table 1**

|  | **Sum sq** | **Mean sq** | **NumDF** | **DenDF** | **F value** | **Pr(>F)** |
| --- | --- | --- | --- | --- | --- | --- |
| Treatment | 550091 | 275046 | 2 | 2 | 0.79 | 0.5593604 |
| Treatment*Sex | 5666678 | 2833339 | 2 | 1552.9 | 8.12 | 0.0003119 |

**Table 2**

|  | **Sum sq** | **Mean sq** | **NumDF** | **DenDF** | **F value** | **Pr(>F)** |
| --- | --- | --- | --- | --- | --- | --- |
| Treatment | 2440687 | 1220344 | 2 | 46.1 | 1.32 | 0.27613 |
| Day | 1382096130 | 1382096130 | 1 | 1060.5 | 1498.90 | <2e^-16^ |
| Treatment*Day | 4803594 | 2401797 | 2 | 1060.5 | 2.60 | 0.07439 |

**Table 3**

|  | **Mean body size (pm^3^)** | **±SE** |
| --- | --- | --- |
| **Females** | 4574 | 30.42 |
| ANC (n=302) | 4392 | 50.62 |
| **FB treatment** (n=260) | 4740 | 51.39 |
| FB1 | 4687 | 87.49 |
| FB2 | 4863 | 106.73 |
| FB3 | 4671 | 70.78 |
| **MB treatment** (n=294) | 4612 | 53.77 |
| MB1 | 4141 | 80.44 |
| MB2 | 4791 | 88.57 |
| MB3 | 4810 | 92.00 |
| **Males** | 1596 | 9.91 |
| ANC (n=318) | 1566 | 15.79 |
| **FB treatment** (n=294) | 1645 | 18.92 |
| FB1 | 1676 | 33.93 |
| FB2 | 1751 | 36.53 |
| FB3 | 1520 | 23.22 |
| **MB treatment** (n=291) | 1577 | 16.49 |
| MB1 | 1454 | 26.36 |
| MB2 | 1635 | 29.05 |
| MB3 | 1609 | 25.75 |

**Table 4**

|  | **Sum sq** | **Mean sq** | **NumDF** | **DenDF** | **F value** | **Pr(>F)** |
| --- | --- | --- | --- | --- | --- | --- |
| Treatment | 1553 | 777 | 2 | 14.99 | 0.12 | 0.88579 |
| Treatment*Sex | 47582 | 23791 | 2 | 1441.59 | 3.75 | 0.02387 |
| Body size | 201221 | 201221 | 1 | 1557.18 | 31.68 | 0.00000002158 |

**Table 5**

|  | **Mean peak fitness (λ_peak_)** | **±SE** |
| --- | --- | --- |
| **Females** | 137 | 30.02 |
| ANC (n=325) | 146 | 48.64 |
| **FB treatment** (n=305) | 138 | 51.80 |
| FB1 | 150 | 85.11 |
| FB2 | 115 | 108.92 |
| FB3 | 149 | 70.46 |
| **MB treatment** (n=317) | 129 | 53.65 |
| MB1 | 135 | 77.65 |
| MB2 | 135 | 86.31 |
| MB3 | 117 | 88.79 |
| **Males** | 301 | 10.19 |
| ANC (n=307) | 299 | 16.49 |
| **FB treatment** (n=286) | 300 | 19.53 |
| FB1 | 298 | 37.01 |
| FB2 | 304 | 36.34 |
| FB3 | 299 | 24.77 |
| **MB treatment** (n=306) | 305 | 16.93 |
| MB1 | 325 | 25.71 |
| MB2 | 311 | 29.53 |
| MB3 | 280 | 27.49 |

**Figure legends**

***Figure 1*** *Female body size (pm^3^) on day 3 & 6 of adulthood. On the x-axis is day of adulthood, and on the y-axis is mean body size (pm^3^). In red is the ANC population, in green FB populations and in yellow MB populations. Error bars represent standard errors.*

**Figure 1**
